# Construction and verification of nomogram prediction model for non-suicidal self-injury in adolescents with depression

**DOI:** 10.1186/s40359-025-02789-8

**Published:** 2025-10-15

**Authors:** Yuehong Gao, Yun Chen, Jiajia Shi, Xiaoli Mao, Jinhong Wang, Jialu He, Hongmei Huang, Xujuan Xu

**Affiliations:** 1 The Fourth People’s Hospital of Nantong, Jangsu, China; 2 Nantong University of Naontong, Jangsu, China; 3 Nursing Research Institute, Affiliated Hospital of Nantong University, Jangsu, China

**Keywords:** Adolescent depression, Non-suicidal self-injury (NSSI), Nomogram, Prediction, Model

## Abstract

**Background:**

Accurate identification of high-risk individuals for NSSI and timely intervention are critical for mitigating self-harm risk. This study aimed to develop a predictive model for NSSI behaviors in adolescents with depression.

**Methods:**

A convenience sample of 596 adolescents with depression was assessed, with 455 assigned to the training and internal validation set and 144 to the external validation set. Nine key predictors were identified through univariate analysis, LASSO regression, and binary logistic regression, including birth mode, history of peer self-harm, parental psychiatric disorders, sleep duration, social life events, self-esteem, psychological resilience, social support, and depression severity. A nomogram-based prediction model was constructed from these factors, with model performance evaluated *via* ROC curves, AUC values, Hosmer-Lemeshow test, and calibration curves. Clinical applicability was determined using decision curve analysis (DCA).

**Results:**

The model exhibited an AUC of 0.880 (*P* < 0.001), with sensitivity of 0.933 and specificity of 0.765. The Hosmer-Lemeshow test confirmed good model fit (χ^2^ = 7.19, *P* = 0.516). Both internal and external validations demonstrated strong discrimination, calibration, and clinical relevance.

**Conclusion:**

The nomogram-based risk model developed in this study effectively predicts NSSI behaviors in adolescents with depression, offering significant scientific and clinical value and warranting further implementation.

**Supplementary Information:**

The online version contains supplementary material available at 10.1186/s40359-025-02789-8.

## Introduction

Non-suicidal self-injury (NSSI) refers to the deliberate, repetitive infliction of harm on one’s body or tissues without suicidal intent. While socially stigmatized, NSSI does not directly result in death but often indicates significant psychological or emotional distress [1]. In Europe and the United States, the prevalence of NSSI among adolescents is notably high. For instance, a study in the United Kingdom found that approximately 20% of adolescents engage in self-harming behaviors [2], while studies in the United States report a prevalence rate ranging from 15–20% [3]. In China, the detection rate of NSSI among adolescents varies between 5.4% and 23.2%, with a significantly higher prevalence of 44.0-61.2% among adolescents suffering from depression [4]. NSSI can transiently increase endogenous opioid levels, leading to temporary feelings of pleasure, which contributes to its addictive nature and the difficulty in treating it [5]. Research indicates that only 50% of young people who engage in self-harm confide in others about their behavior [6]. Additionally, the onset of NSSI is not fixed, and when adolescents choose not to disclose their distress, it becomes challenging for family members to recognize abnormal behaviors due to a lack of constant monitoring. These recurrent self-injurious behaviors are outward manifestations of internal pain, and failure to identify and address them early significantly elevates the risk of suicide [7]. The presence of NSSI in depressed individuals increases their suicide risk by more than sevenfold [8]. Given the ongoing rise in suicide rates, NSSI is increasingly recognized as a critical predictor of future suicide risk, and its potential threat must not be overlooked [9]. Preventing NSSI in adolescents with depression is critical for mitigating the progression to suicidal behavior. Early identification of high-risk individuals and the provision of targeted interventions are essential for effectively preventing NSSI and its escalation into more severe outcomes, including suicide. Zuo Sweet’s study identified several predictors of NSSI in adolescents with depression, including age, education level, childhood trauma, depression severity, and peer rejection [10]. In contrast, Yang Yuping’s research emphasized the impact of factors such as poor family environment, impulsivity, anxiety, and dysfunctional interpersonal relationships [11]. International studies have also highlighted the significant role of parenting style in the onset of self-injurious behavior in depressed adolescents, with negative parenting contributing to the likelihood of such behaviors [12]. Furthermore, childhood physical abuse has been linked to the development of depression, personality disorders, and other psychological issues that increase the risk of NSSI [13]. However, relying on a single factor or a limited set of factors is inadequate for accurately predicting NSSI in adolescent patients with depression. To improve prediction accuracy, it is necessary to assess the predictive weight of each factor and develop a reliable, quantitative risk assessment tool that can accurately identify individuals at high risk for NSSI. This study employed a multi-center cross-sectional survey to explore the factors influencing self-injurious behavior in adolescents with depression and to construct a predictive model aimed at identifying high-risk individuals. By doing so, it aims to provide early interventions that can prevent NSSI and support the overall physical and mental well-being of adolescents.

## Materials and methods

### Study participants

The study involved adolescents diagnosed with depression who were treated at Nantong Fourth People’s Hospital, Nanjing Brain Hospital, and Suzhou Guangji Hospital between December 2022 and July 2023. Inclusion criteria were as follows: (1) age between 11 and 18 years [14]; (2) meeting the diagnostic criteria for depression, including a persistently depressed mood or reduced interest in activities lasting at least two weeks, accompanied by difficulty concentrating, feelings of worthlessness or excessive guilt, and hopelessness, in accordance with the ICD-11 Classification of Mental and Behavioral Disorders: Clinical Description and Diagnostic Points [15]; (3) sufficient communication skills, including the ability to read and express oneself clearly. Exclusion criteria included: (1) diagnosis of other mental disorders; (2) history of alcohol or drug abuse; (3) severe physical illnesses; (4) hearing or vision impairments; (5) history of suicidal behaviors (e.g., jumping from heights, hanging, ingesting lethal doses of drugs, or other clear self-injurious behaviors intended to cause death).

### Sample size

Model construction was based on the minimum number of observations for both outcome types in a dichotomous outcome setting. The literature indicates a 51% prevalence of NSSI among adolescents with depression. With 10 variables included in the model, the sample size requirement was calculated to be at least 205 cases (with a minimum of 100), adhering to the ten events per variable (EPV) principle. Anticipating a 10% missing data rate and potential invalid questionnaires, 228 cases were included for the training and internal validation sets, with 70% allocated to the training set and 30% to the internal validation set. External validation required an additional 68 cases, matching the sample size of the internal validation group. Therefore, the total planned sample size was 296 cases. Ultimately, 596 patients were included in the study.

### Methods

#### General information survey

A self-administered questionnaire was utilized to collect data on age, sex, whether there is self-injury, being a single child, relationships and love experiences, sleep duration, online activity duration, parental education, cohabitation status, parental marital status, parental relationships, residence, and family income.

#### The behavioral functional assessment scale for non-suicidal self-injury in adolescents

It was initially developed by Whitlock [16] and later Sinicized and revised by Zhang L.J [17]. It is used to evaluate the frequency, purpose, mode and influence of NSSI, so as to understand whether adolescents have NSSI and the specific situation of NSSI.

#### The eysenck personality questionnaire (EPQ)

It was initially developed by Donald E [18], and subsequently sinicized and revised by Gong Y.X [19]. It was employed to assess the personality dimensions of the patients. The questionnaire consists of 88 questions and includes three personality subscales: extraversion (E), neuroticism (N), and psychoticism (P), and a validity subscale (L). The cumulative scores of each subscale were transformed into standardized T-scores based on age and gender norms. The Cronbach’s α-coefficient is 0.81.

#### The family intimacy and adaptability scale (Family adaptation and cohesion evaluation scales II, FACES II)

It was developed by Olson in 1982 [20], and subsequently sinicized and revised by Fei L.P [21]. It encompasses two subscales: ‘Family Intimacy’ (the emotional interconnectedness of individual family members) and ‘Family Adaptability’ (the capacity of individual members to make appropriate adjustments in response to varying family dynamics and stages). FACES II is a tool for evaluating family functioning by measuring intimacy and adaptability. The scale demonstrated satisfactory reliability and validity, with Cronbach’s α-coefficient of 0.85.

#### Self-esteem scale (Rosenberg self—esteem scale, SES)

It was initially developed by Rosenberg M [22], and later sinicized and revised by Ji C.F [23]. This scale examines an individual’s positive or negative self-attitude. Comprising ten questions graded on a four-level scale from “very consistent” to “very inconsistent,” the ranking reflects the level of self-esteem, with higher scores indicating elevated self-esteem. The scale’s robust internal consistency is characterized by Cronbach’s α-coefficient of 0.90.

#### Conner–davidson resilience scale (CD-RISC10)

CD-RISC10 was derived from the work of Campbell-Sills and Stein [24], later sinicized and revised by Dai X.Y [25]. It serves as a concise iteration of the CD-RISC, gauging an individual’s resilience and adaptive capacity when confronted with adversity. The scale, involving ten self-rated items, employs a 5-point Likert scale from 0 (“not true at all”) to 4 (“true nearly all the time”). The resulting scores, ranging from 5 to 50, correlate with higher levels of resilience. The CD-RISC10 exhibits robust internal reliability (Cronbach’s α = 0.89).

#### Childhood trauma questionnaire (CTQ)

A simplified adaptation of the CTQ, initially revised by Bernstein et al. [26]. and later by Zhao. X.F [27]. in 2003 assesses the abuse and neglect experienced during childhood. The CTQ comprises 28 items categorized into five subscales: emotional abuse, physical abuse, sexual abuse, emotional neglect, and physical neglect. The Cronbach’s α-coefficient is 0.82.

#### Adolescent social support scale (social support rate scale, SSRS)

According to Xiao.‘s social support theory [28], Yei and Dai compiled a social support scale for adolescents in 2008 [29]. This scale comprises three dimensions: subjective social support, objective social support, and utilization of social support. These dimensions evaluate the social support available to adolescents and their interaction with this support system. The scale encompasses 17 items, each rated on a five-point scale, with “Meets” receiving 5 points, “Somewhat Meets” = 4 points, “Unsure” = 3 points, “Somewhat” = 2 points, and “Not sure” = 1 point. The scale’s internal consistency, Cronbach’s α-coefficient is 0.82.

#### Adolescent society life events scale (ASLEC)

Compiled by Liu [30], this scale assesses the frequency and intensity of stressful life events among adolescents, particularly middle school and college students. The correlation coefficients between individual event scores and the total score range from 0.24 to 0.57, averaging 0.45. This scale demonstrates satisfactory reliability and validity, with Cronbach’s α-coefficient of 0.85.

#### Barratt impulsivity scale (BIS-11)

The 11th edition of the Impulsivity Scale was compiled by Barratt et al. and revised by Zhou L. et al. [31]. was used to assess the impulsivity trait, which has a Cronbach’s α-coefficient of 0.76, retest reliability of 0.85, and a good structural validity index. The questionnaire consists of 26 items covering three dimensions, namely attentional impulsivity, locomotor impulsivity and unplanned impulsivity, and is scored on a 4-point scale (from 1 ‘never’ to 4 ‘all the time’), with higher scores indicating greater impulsivity.

#### Self-rating anxiety scale (SAS)

SAS was derived from the work of Zung W.W [32]. It is a unidimensional scale with 20 items, including five reverse-scored questions. The sum of the scores for each item was multiplied by 1.25 and rounded to the nearest whole number to obtain the standardized anxiety score. A 4-point Likert scale was used, with one indicating none or very little time, four indicating most or all of the time, and four indicating most or all of the time. The total standardized score ranges from 25 to 100, with higher scores indicating greater levels of anxiety. Higher scores indicate greater levels of anxiety. The scale has a cut-off for anxiety, with < 50 indicating no anxiety, 50–59 indicating mild anxiety, 60 − 59 indicating moderate anxiety, and ≥ 70 indicating severe anxiety. The Cronbach’s α-coefficient is 0.86.

#### Self-rating depression scale (SDS)

The scale was developed by Zung to quantify the amount of money received. Measure the severity and treatment of depressive state Changes in therapy (zung 1971). There are 20 items on the scale. Using a 4-level scoring method, each of the 20 items. If you add up all the scores, you get a rough score; Multiply by the rough parts. Take the integer part after 1.25. Obtained standard Points. The higher the standard score, the more severe the depression. According to the results of the Chinese norm, the SDS standard is divided into 53 points, 53 to 62 points for mild depression, 63 to 72 was classified as moderate depression, and ≥ 72 was classified as severe depression. The Cronbach’s α-coefficient is 0.86.

### Statistical analysis

#### Analytical methods

Two individuals entered all survey data into Excel software. Missing data included four instances concerning parental relationships, one related to parental mental health, six regarding family income, and six related to delivery mode. Variables with less than 5% missing data were not imputed. Statistical analysis was performed using SPSS 25.0 and R language 4.1.1. Normally distributed continuous variables were expressed as means and standard deviations, with comparisons made using an unpaired two-tailed t-test. Categorical data were presented as frequencies and percentages, and comparisons were conducted using χ^2^ or Fisher’s exact tests. A significance level of *P* < 0.05 was considered statistically significant.

#### Variable selection and prediction model establishment

Initial variable screening was performed using univariate analysis. Subsequently, LASSO regression was applied to identify potential predictors. The factors selected through LASSO regression were then incorporated into binary logistic regression for further refinement. Only variables that showed statistical significance (*P* < 0.05) were retained for inclusion in the risk prediction model. The dataset was randomly divided into training and validation sets, with 70% allocated to model development. Model performance was assessed using multiple metrics, including the Area Under the Curve (AUC), which measured the model’s ability to discriminate between different outcomes. Calibration of the model was evaluated using ROC calibration curves, and the goodness-of-fit was tested with the Hosmer-Lemeshow test. Decision Curve Analysis (DCA) was employed to assess the clinical utility of the model across various threshold probabilities.

#### Internal and external validation of the model

For internal validation, 30% of the data was reserved, and additional data from other hospitals were conveniently sourced for external validation. The model’s discriminability, calibration, and clinical utility were evaluated using the AUC, Hosmer-Lemeshow goodness-of-fit test, calibration curve, and DCA, respectively.

### Data collection

1. Setting and Participant Selection: Data were collected in two distinct environments: outpatient and inpatient settings. For outpatient data collection, participants were selected based on their preference for a quiet, comfortable, and well-lit single-room clinic environment following their consultation. In the ward setting, patients were chosen based on their stable mood and preference for a quiet, comfortable, and private meeting room.
2. Participant Recruitment and Consent: Adolescent patients diagnosed with depression were recruited for this study. Before participation, both adolescent patients and their parents or legal guardians were provided with detailed information about the study’s purpose, content, and significance. Informed consent was obtained from all participants, and written consent was also secured from the legal guardians of minors. A signed informed notification letter was given, outlining the scope of the study and offering guidance on completing the questionnaire.
3. Questionnaire Administration: A standardized approach, employing consistent instructions and terminology, was used to guide adolescent patients with depression through the questionnaire process. Additional explanations and clarifications were provided to participants to address any queries or uncertainties during the completion of the questionnaire.

### Ethical considerations

This paper adheres to the ethical principles set forth in the Declaration of Helsinki by the World Medical Association. The ethical considerations guiding this study encompass:

#### Informed consent

Prior to participation, all subjects provided informed consent. They were informed comprehensively about the study’s objectives, procedures, potential risks, and their right to withdraw at any time without repercussion.

#### Confidentiality

Strict measures were implemented to safeguard participant confidentiality. All data collected were anonymized and securely stored to prevent unauthorized access.

#### Beneficence and non-maleficence

The study aimed to maximize benefits for participants while minimizing potential harm. Stringent measures were taken to mitigate risks during data collection and analysis.

#### Research design and approval

The study was approved by the Ethics Committee of the Fourth People’s Hospital of Nantong City (approval no.: 2022-Ko37).

#### Conflict of interest disclosure

The researchers affirm no conflicts of interest that could have influenced the study’s outcomes or interpretations.

#### Participant treatment

Participants were treated with utmost respect and dignity throughout the study. Their voluntary participation was paramount, and efforts were made to ensure their comfort and safety. Participants over 16 years old signed the informed consent form after obtaining their consent. Participants under the age of 16 shall sign the informed consent form after obtaining the consent of themselves, their parents or legal guardians.

#### Regulatory compliance

This study adhered to all relevant laws and regulations governing research involving human subjects in China.

## Results

### Univariate analysis of influencing factors

The study included 455 participants, consisting of 131 males and 324 females, aged 11–18 years (mean age: 14.96 ± 1.88). Among them, 272 had engaged in NSSI behavior in the past year, while 183 had not. Univariate analysis identified significant factors, such as sex, delivery mode, relationship status, experience of heartbreak, history of peer NSSI, parental psychiatric history, sleep duration, online time, parental education, cohabitation status, parental relationship, parents’ marital status, monthly household income, and scores on various scales including FACES II, SSRS, SES, CD-RISC10, ASLEC, SDS, SAS, and the Barratt scale (*P* < 0.05) (Multimedia Appendix 1).

### LASSO regression identifies potential predictors

**Fig. 1:**
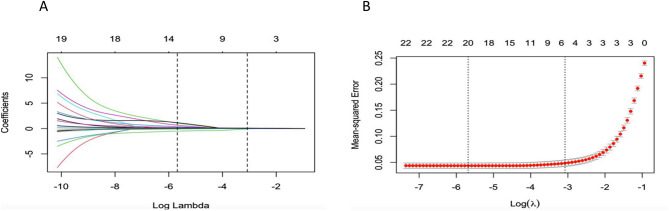
The variable filtering process of the Lasso regression. (**A**) Lasso coefficient profiles of the candidate features; (**B**) the selection of optimal parameters (lambda) by tenfold cross-validation. A coefficient profile plot was generated across the log (lambda) sequence

Following the univariate analysis, 22 variables were included for characteristic variable screening in LASSO regression. The LASSO regression analysis was performed using the ‘cv.glmnet’ package in R4.1.1. By adjusting the penalty function (λ), the coefficients of the model’s variables were progressively compressed until those with minimal effect on the results were reduced to zero. Each independent variable was represented by a corresponding curve, illustrating the changes in the coefficients of each variable during the penalization process. The optimal lambda values were determined using five-fold cross-validation with the ‘cv.glmnet’ function (Fig. 1A). Independent variables were then filtered based on the optimal lambda value (Fig. 1B). The ‘predict’ function identified 13 potential predictors at the optimal lambda value, which included gender, delivery mode, history of peer self-harm, history of parental psychiatric illness, academic performance, sleep duration, personality traits, level of family closeness and adjustment, social life events, psychological resilience, self-esteem, social support, and depression.

### Binary logistic regression to determine variables for inclusion in the model

Based on the results of the LASSO regression analysis, 13 potential predictors were included in a binary logistic regression to identify the variables ultimately retained in the model. A multivariate stepwise logistic regression analysis further refined the predictors of self-injury, narrowing them down to nine variables (Table 1). These predictors included delivery mode, history of peer NSSI, parental psychiatric history, sleep duration, social life events, self-esteem, psychological resilience, social support, and depression (*P* < 0.05).

**Table 1:** Predictors of NSSI risk in adolescents with depression

Intercept and variables	Prediction model				
	β	z value	OR	*P* value	95% CI
Sex	0.497	0.333	2.229	0.135	0.856–3.155
Delivery mode	1.074	0.300	12.768	0.000	1.624–5.272
History of peer NSSI	0.658	0.334	3.880	0.049	1.003–3.720
Parental psychiatric history	2.145	0.824	6.781	0.009	1.700-42.916
Score	-0.179	0.203	0.781	0.377	0.562–1.244
Sleep time	-0.746	0.200	13.880	0.000	0.320–0.702
EPQ	0.203	0.225	0.818	0.366	0.789–1.903
FACES II	-0.005	0.008	0.447	0.504	0.980–1.010
SSRS	-0.060	0.011	29.530	0.000	0.921–0.962
CD-RISC	-0.051	0.012	18.314	0.000	0.928–0.973
SES	-0.157	0.031	25.014	0.000	0.804–0.909
ASLEC	0.053	0.009	34.217	0.000	1.036–1.073
SDS	0.039	0.015	6.596	0.010	1.009–1.070

### Construction of the risk prediction model

**Fig. 2:**
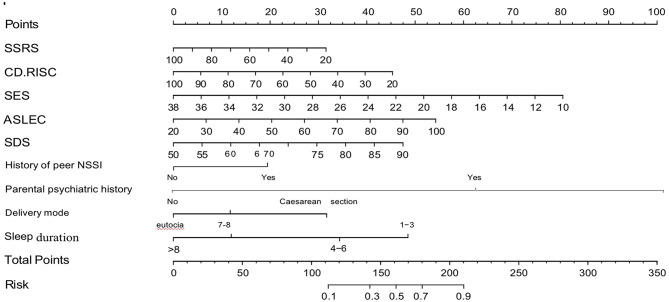
Nomogram prediction model for non-suicidal self-injury in adolescents with depression

**Fig. 3:**
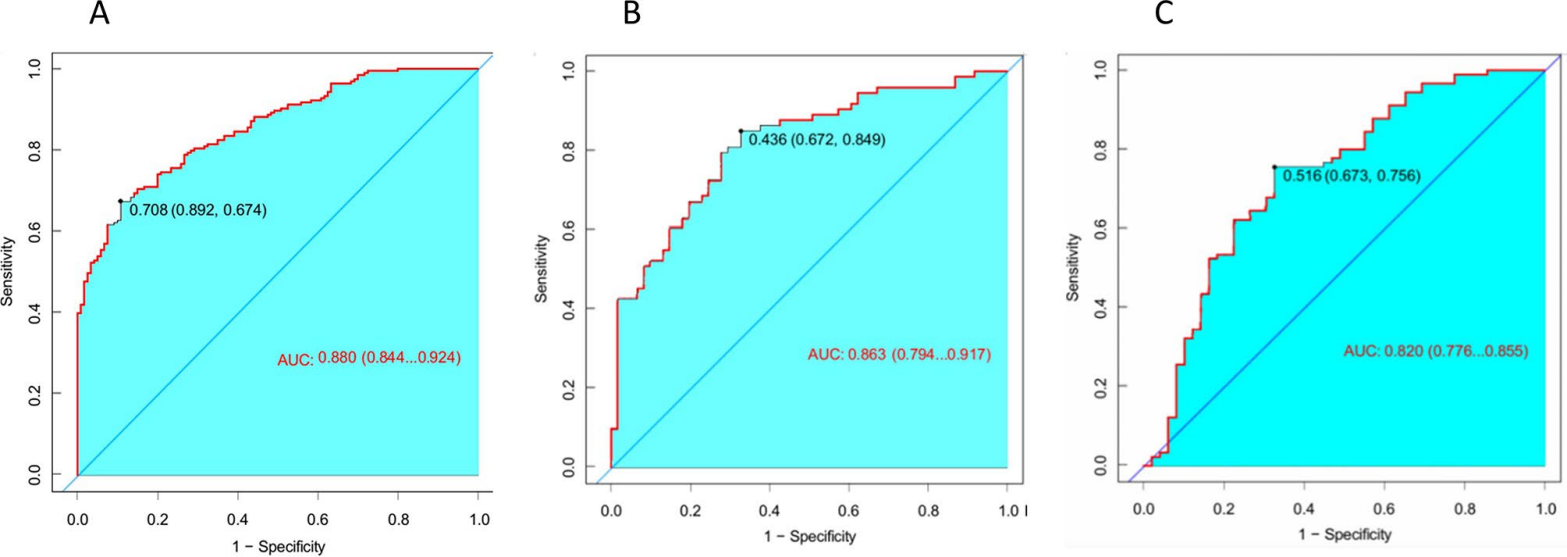
ROC curve of NSSI risk prediction model; (**A**) ROC curve of the training set risk prediction model; (**B**) ROC curve of internal risk set risk prediction model; (**C**) ROC curve of external risk set risk prediction model

**Fig. 4:**
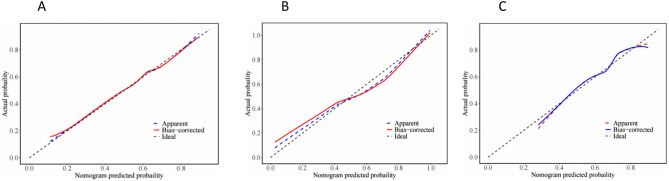
Calibration curve of risk prediction model. (**A**) Calibration curve of the training set risk prediction model; (**B**) Calibration curve of the internally validated set risk prediction model; (**C**) Calibration curve of external validation set risk prediction model. Note: The Y-axis represents actual NSSI occurrence and the X-axis represents projected NSSI risk. The solid red line represents the perfect prediction of the ideal model. The blue dashed line describes the performance prediction graph and represents a cross-validation graph of a ten-fold model construction

The clinical sample data were randomly divided into a training set (70%) and a validation set (30%), with participants classified into self-injurious and non-self-injurious groups based on the presence or absence of NSSI. A nomogram risk prediction model for NSSI was developed using the nine predictors identified through univariate, LASSO regression, and dichotomous logistic regression analyses (Fig. 2). The nomogram consists of scores for each predictor: delivery mode, peer self-injury history, parental psychiatric history, sleep duration, social life events, self-esteem, psychological resilience, social support, and self-rated depression. The total score, derived from the sum of individual scores, corresponds to the predicted probability of NSSI occurrence. The model’s performance, assessed by the area under the ROC curve, yielded a value of 0.880 (*P* < 0.001), indicating strong discriminative ability (Fig. 3A). The optimal cut-off value, determined by the maximum Youden index, was validated by the Hosmer-Lemeshow test (χ2 = 7.19, *P* = 0.516), yielding a maximum Youden index of 0.668, a specificity of 0.765, and a sensitivity of 0.933. A calibration plot demonstrated excellent calibration, with data point connectors closely aligning with the diagonal line (Fig. 4A). DCA for the training set revealed significant net benefit across nearly all risk threshold probabilities, particularly within the 0.1-1.0 range (Multimedia Appendix 2 A).

For example, in an adolescent with depression, where the mode of delivery was normal (0 points), peers had a history of self-harm (20 points), parents had no psychiatric history (0 points), sleep duration was 4–6 h daily (35 points), social life events scored 90 (47 points), self-esteem was 38 (0 points), psychological resilience was 50 (28 points), social support was 60 (17 points), and self-rated depression was 80 (36 points), the total score was 183, corresponding to a predicted probability of 70%.

### Internal validation of the prediction models

30% of the data were allocated for internal validation of the nomogram risk prediction model, yielding an area under the ROC curve of 0.863 (*P* < 0.001) in the internal validation set (Fig. 3B). The Hosmer-Lemeshow goodness-of-fit test revealed a χ2 of 11.366 (*P* = 0.182), with a maximum Youden index of 0.986, specificity of 0.994, and sensitivity of 0.992. Calibration curves for the internal validation set, plotted after 1000 replicate samples, demonstrated a strong alignment with the diagonal line, indicating robust predictive performance for NSSI (Fig. 4B). DCA for the internal validation set showed a significant net gain across most risk threshold probabilities, particularly within the 0.25–0.95 range (Multimedia Appendix 2B).

### External validation of the prediction model

Adolescent patients with depression, admitted to Nanjing Brain Hospital and Suzhou GuangJi Hospital between December 2022 and August 2023, were selected as the external validation data set. The nomogram’s ROC curve in the external validation set yielded an area of 0.820 (Fig. 3C), with a maximum Youden index of 0.523, sensitivity of 0.645, and specificity of 0.878. The Hosmer-Lemeshow test for the external validation set produced a χ2 of 9.466 (*P* = 0.305). The calibration curve for the external validation set also demonstrated strong performance in predicting NSSI (Fig. 4C). The DCA for the external validation set revealed that the nomogram risk prediction model provided a significant net benefit across most risk threshold probabilities, particularly within the 0.45–0.85 range (Multimedia Appendix 2 C).

## Discussion

This study developed a risk prediction model incorporating nine key variables: depression severity, sleep duration, social life events, parental history of mental illness, peer history of NSSI, mode of birth, mental resilience, self-esteem, and social support. All these factors demonstrated significant associations with NSSI behavior, offering valuable insights into the multi-dimensional influences on NSSI.

Previous research has extensively examined the strong link between depression severity and the tendency for NSSI in adolescents, with the consensus that depression severity is a major determinant of NSSI incidence [13, 33]. Further studies have revealed a decreased serotonin receptor binding index in the frontal lobes of depressed individuals with NSSI compared to those without, suggesting that neurobiological differences may contribute to NSSI behavior [34]. Additionally, elevated levels of proinflammatory cytokines have been observed in depressed patients with NSSI, with these individuals often displaying more aggressive personality traits. Analysis indicates a positive correlation between proinflammatory cytokine levels and impulsivity [35].

Extensive research has highlighted a marked reduction in sleep duration as a key predictor of NSSI behavior [36, 37]. Furthermore, studies have shown significant correlations between nightmares, irregular sleep, and NSSI. This study reinforces the independent effect of sleep duration on NSSI in adolescents with depression, although it did not explore sleep patterns in detail. Future research could focus on the specific impact of sleep patterns on NSSI behaviors, offering more targeted intervention strategies.

This study also underscores the significant role of social life events in the development of NSSI. Negative life events, such as academic stress, interpersonal conflicts, and family issues, are widely recognized as major triggers for NSSI. Adolescents, with limited coping mechanisms and adaptive skills, are particularly vulnerable to intense negative emotions in the face of challenges, often resorting to NSSI as a means of emotional release [38]. It is essential for families, schools, and society at large to prioritize adolescent mental health, proactively minimize exposure to adverse life events, and provide the necessary support to foster healthy development.

Mental disorders, including schizophrenia, mood disorders, anxiety, and related conditions, not only lead to abnormalities in thinking, attention, and behavior—such as cognitive dysfunction, inattention, and impulsivity—but also significantly impair an individual’s functioning across various domains, including personal, family, social, and occupational spheres [39]. Parents with mental disorders can undoubtedly negatively impact the family environment, a finding widely corroborated by both domestic and international studies [40–41].

Research indicates that adolescents who have witnessed a relative’s suicide or self-injury are more likely to imitate these behaviors as a way of coping with distress and frustration [42]. Qualitative interviews with adolescents suffering from depression and NSSI have revealed a tendency to mimic peers’ NSSI behaviors after exposure to such acts [43]. However, research examining this phenomenon, both domestically and internationally, remains scarce, and there is a lack of robust theoretical frameworks to support these findings.

Infants born *via* cesarean section are subjected to unique hormonal, physiological, bacterial, and medical influences that can subtly alter their physiological state [44]. Moreover, multiple studies have established a connection between cesarean section delivery and a variety of mental and behavioral issues, including attention deficit hyperactivity disorder, autism spectrum disorder, and other behavioral problems in children [45]. The use of general anesthesia during cesarean sections may result in neurotoxic substances and oxytocin dysregulation, factors closely linked to the development of autism in children [46]. Our exploration of the effect of delivery mode on NSSI behavior in adolescents with depression suggests that cesarean section is a significant factor influencing NSSI in this group. Given the paucity of research in this area, more comprehensive studies on cesarean delivery and its impact on the mental and behavioral health of offspring would provide a more scientific basis for the rational use of cesarean sections and better safeguard the physical and mental well-being of children.

Individuals with higher psychological resilience are better equipped to employ effective coping strategies when faced with adversity, utilizing surrounding resources more flexibly and intelligently, which in turn helps prevent self-harm [47]. In the family context, a strong parent-child relationship offers crucial support, not only providing social backing and resilience in the face of setbacks but also aiding children in developing a sense of self-worth. When engaging with children, it is essential to listen attentively and understand their inner world, fostering healthy growth and strengthening psychological resilience. Thus, enhancing adolescents’ psychological resilience holds promise as a more effective strategy for preventing and reducing NSSI behaviors.

Individuals with high self-esteem typically possess a positive self-image, confidence, and a proactive approach to challenges. In contrast, individuals with low self-esteem tend to harbor negative self-perceptions and lack confidence, making them more likely to resort to escape mechanisms or self-harm in the face of difficulties [48]. Improving adolescent self-esteem can be achieved through various approaches, such as cultivating their interests and helping them set and achieve goals, thereby reinforcing their sense of value and accomplishment.

Social support, encompassing both emotional and material assistance, plays a pivotal role in an individual’s well-being. Prolonged lack of social support can lead to emotional disorders, interpersonal tension, and cognitive distortions, significantly increasing the risk of NSSI [49]. Rebuilding and enhancing social support systems is vital in preventing NSSI among adolescents. At the societal level, an integrated system involving hospitals, communities, schools, and families should be established, with a focus on at-risk individuals. By providing emotional support and addressing practical issues, adolescents can feel valued and respected. Additionally, offering accessible communication channels ensures that they receive timely assistance and guidance when facing confusion or challenges, promoting overall physical and mental well-being.

## Advantages and limitations

This research has a certain degree of advantages. First, unique choice of research subject: In today’s society, NSSI behaviours have attracted much attention, and adolescents, as a major group among them, are even a hotspot for research. However, this study uniquely focuses on the special group of adolescents with depression and explores in depth the factors influencing their NSSI behaviours. This unique research perspective not only enriches the research content in related fields, but also provides valuable clues for understanding and solving the NSSI problems of adolescents with depression. Second, Deepening of research content: Unlike previous studies that mainly focused on the analysis of NSSI influencing factors, this study further explored in depth the independent influencing factors on the occurrence of NSSI in adolescent depressed patients, and successfully constructed a risk prediction model. This model can accurately predict the probability of occurrence of NSSI behaviours in adolescent depression patients, providing a scientific basis for clinical intervention and risk assessment. Finally, Comprehensive innovation of research methodology: This study adopted a variety of advanced statistical methods, such as single-factor analysis, LASSO regression analysis and binary logistic regression equation analysis, which effectively avoided the problems of covariance and overfitting and ensured the scientificity and reliability of the model. This study has several limitations. Firstly, the data collection relied on patients completing questionnaires, which could introduce risks such as patients avoiding certain questions or recall bias. Additionally, due to individual differences among the study samples and the inherent limitations of the questionnaire, there may be generalization and bias in the results. Future research should focus on longitudinal follow-up studies, with regular check-ins of participants and the detailed recording of relevant information at various time points. Moreover, future studies should incorporate imaging and laboratory indicators to explore the biological mechanisms underlying NSSI from multiple dimensions, providing more comprehensive and convincing scientific evidence to enhance the quality and value of research.

## Conclusions

This study employed a combination of univariate analysis, LASSO regression, and binary logistic regression to identify nine optimal predictors for self-injury and construct a nomogram to predict NSSI risk. These predictors include mode of delivery, peer self-injury history, parental history of mental illness, sleep duration, social life events, self-esteem, psychological resilience, social support, and depression severity. The nomogram risk prediction model demonstrated strong performance and effectively predicts NSSI occurrences in adolescents with depression, offering both scientific validity and practical insights. It provides theoretical support for accurately screening high-risk populations, implementing effective interventions, and preventing NSSI. External validation confirmed that the model possesses excellent discrimination and calibration, making it a promising tool for wider application and further research.

## Electronic supplementary material

Below is the link to the electronic supplementary material.

Supplementary Material 1

Supplementary Material 2

## Data Availability

This statement will replace any statement written within the manuscript and is the one that we will publish. For example, “Sequence data that support the findings of this study have been deposited in the European Nucleotide Archive with the primary accession code PRJWB13140”, or “Data is provided within the manuscript or supplementary information files”. Find some help on our Data availability statements page.
