# Supplementary material for: Construction and verification of nomogram prediction model for non-suicidal self-injury in adolescents with depression
Source: BMC Psychol. 2025 Oct 15;13:1153. doi: 10.1186/s40359-025-02789-8 (PMC12522271; doi:10.1186/s40359-025-02789-8)
Supplement: Supplementary file 1 — Supplementary Material 1 [file 40359_2025_2789_MOESM1_ESM.docx]

Multimedia Appendix 1 Univariate analysis of NSSI related factors in adolescents with depression

| Sports event | | NSSI | | test statistic *P* | |
| --- | --- | --- | --- | --- | --- |
|  |  | Yes（n=272） | No（n=183） |  |  |
| Sex n(%) | Male | 62（22.8) | 69（37.7) | 11.864^2)^ | <0.001 |
|  | Female | 210（77.2) | 114（62.3) |  |  |
| Delivery mode n(%) | Eutocia | 78（42.9) | 104（57.1) | 27.563^2)^ | <0.001 |
|  | Caesarean Section | 147(55.1) | 120（44.9) |  |  |
| Being an single child n(%) | Yes | 132（48.5) | 79（43.2) | 1.264^2)^ | 0.261 |
|  | No | 140（51.5) | 104（56.8) |  |  |
| Love n(%) | Yes | 75（27.6) | 29（15.8) | 8.532^2)^ | 0.003 |
|  | No | 197（72.4) | 154（84.2) |  |  |
| Be out of love n(%) | Yes | 45（16.5) | 16（8.7) | 6.286^2)^ | 0.012 |
|  | No | 227（83.5) | 167（91.3) |  |  |
| History of peer NSSI n(%) | Yes | 120（44.1) | 46（25.1) | 17.008^2)^ | <0.001 |
|  | No | 152（55.9) | 137（74.9) |  |  |
| Parental psychiatric history n(%) | Yes | 26（9.6) | 4（2.2) | 6.454^2)^ | 0.008 |
|  | No | 245（90.4) | 179(97.8) |  |  |
| Score n(%) | Talented | 11（4.0) | 12（6.6) | -2。828^3)^ | 0.005 |
|  | Favorable | 75（27.6) | 65（35.5) |  |  |
|  | Ordinary | 117（43.0) | 77（42.1) |  |  |
|  | Poor | 69（25.4) | 29（15.8) |  |  |
| Sleep duration n(%) | 3-4 hours | 89（32.7) | 12（12) | -7。026^3)^ | <0.001 |
|  | 5-6 hours | 149（54.8) | 80（37.7) |  |  |
|  | 7-8 hours | 23（8.5) | 69（43.7) |  |  |
|  | >8 hours | 11（4.0) | 22（6.6) |  |  |
| Online activity duration n(%) | <1 hours | 50（18.3) | 65(35.5) | -6.564^3)^ | <0.001 |
|  | 1-3 hours | 77（28.3) | 64（35.0) |  |  |
|  | 4-6 hours | 83（30.5) | 29（15.8) |  |  |
|  | >8 hours | 62（22.8) | 25（13.7) |  |  |
| Parental education n(%) | Have a college degree or above | 46（16.9) | 43（23.5) | -1.486^3)^ | 0.138 |
|  | One of the highest education high school | 87（32.0) | 56（30.6) |  |  |
|  | Both primary or junior high schools | 139（51.1) | 84（45.9) |  |  |
| Co-inhabitant status n(%) | parent | 113（41.5) | 106（57.9) | -3.338^3)^ | 0.001 |
|  | father | 42（15.4) | 20（10.9) |  |  |
|  | mother | 72（26.5) | 39（21.3) |  |  |
|  | other | 45（16.5) | 18（9.8) |  |  |
| Parents' marriage status n(%) | In marriage | 166（61.0) | 148（80.9) | -4.312^3)^ | <0.001 |
|  | Divorced and never remarried | 44（16.2) | 12（6.6) |  |  |
|  | Divorced and remarried | 51（18.8) | 20（10.9) |  |  |
|  | Be bereaved of one's spouse | 10（3.7) | 3（1.6) |  |  |
|  | Death of both parents | 1（0.4) | 0（0) |  |  |
| Parents relationships n(%) | intimate | 46（17.1) | 61（33.5) | -6.601^3)^ | <0.001 |
|  | good | 61（22.7) | 64（35.1) |  |  |
|  | fair | 106（39.4) | 53（29.1) |  |  |
|  | poor | 56（20.8) | 4（2.2) |  |  |
| Family income n(%) | <$2000 | 4（1.5) | 4（2.2) | -2.627^3)^ | 0.009 |
|  | $2000-$5000 | 63（23.4) | 32（17.7) |  |  |
|  | $5000-$10000 | 122（45.4) | 70（38.9) |  |  |
|  | $10000-$20000 | 56（20.8) | 50（27.8) |  |  |
|  | >$20000 | 24（8.9) | 24（13.3) |  |  |
| Residence n(%) | city | 135（49.6) | 95（51.9) | 0.228^2)^ | 0.633 |
|  | village | 137（50.4) | 88（48.1) |  |  |
| EPQ n(%) | neuroticism | 235（86.4) | 141（77) | -3.016^3)^ | 0.002 |
|  | psychoticism | 27（9.9) | 9（4.9) |  |  |
|  | Introversion and extroversion | 6（2.2) | 18（9.8) |  |  |
|  | Simple quality | 4（1.5) | 15（8.2) |  |  |
| Age m±sd | | 14.84±1.914 | 14.94±1.922 | -1.191^1)^ | 0.849 |
| FACES II m±sd | | 73.92±19.965 | 81.13±20.493 | 3.738^1)^ | <0.001 |
| CTQ m±sd | | 54.71±38.419 | 55.07±16.993 | -0.136^1)^ | 0.892 |
| SSRS m±sd | | 53.11±16.244 | 61.68±17.273 | 5.378^1)^ | <0.001 |
| CD-RISC m±sd | | 58.22±18.063 | 64.34±15.638 | 3.842^1)^ | <0.001 |
| SES m±sd | | 20.75±4.862 | 24.64±5.748 | 7.538^1)^ | <0.001 |
| ASLEC m±sd | | 59.60±15.869 | 52.01±14.434 | -5.188^1)^ | <0.001 |
| SDS m±sd | | 70.22±11.417 | 63.81±9.631 | -6.447^1)^ | <0.001 |
| SAS m±sd | | 73.66±7.504 | 53.78±9.240 | -25.201^1)^ | <0.001 |
| Battatt m±sd | | 88.52±9.021 | 63.08±9.987 | -28.202^1)^ | <0.001 |
