# Supplementary material for: Construction and verification of nomogram prediction model for non-suicidal self-injury in adolescents with depression
Source: BMC Psychol. 2025 Oct 15;13:1153. doi: 10.1186/s40359-025-02789-8 (PMC12522271; doi:10.1186/s40359-025-02789-8)
Supplement: Supplementary file 2 — Supplementary Material 2 [file 40359_2025_2789_MOESM2_ESM.docx]

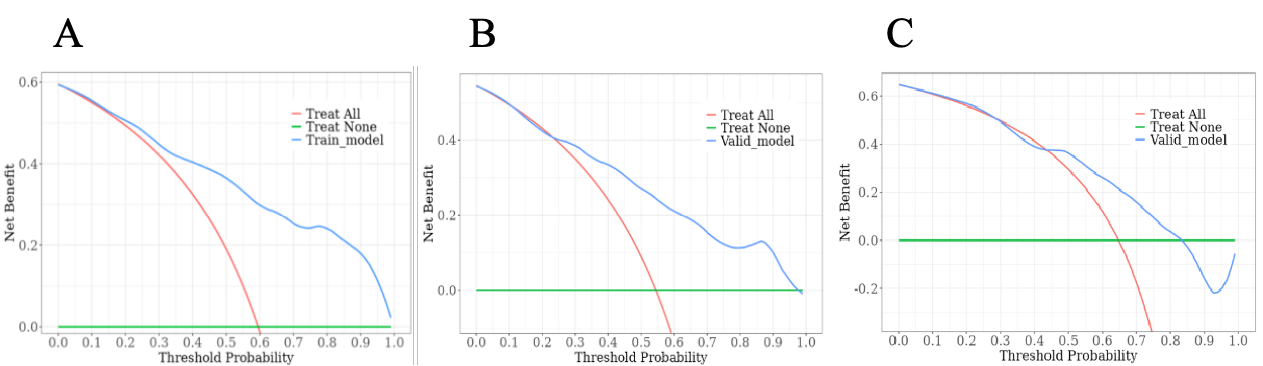


Multimedia Appendix 2. Decision analysis curve of risk prediction model. (A)Risk prediction model training set decision analysis curve;(B)Decision analysis curve of internal validation set of risk prediction model; (C)Decision analysis curve of external validation set of risk prediction model.

Note: The horizontal and vertical axes indicate threshold rates and net benefits, respectively, and the lines between the horizontal and vertical axes indicate benefits for different predictors. The green line represents all patients without NSSI, the red curve represents all patients with NSSI, and the blue curve represents the performance of the risk map.
